# Catalytic performance of α-MnO_2_ nanorods on the degradation of rhodamine B using chlorine dioxide as an oxidant

**DOI:** 10.1039/d5ra07092a

**Published:** 2025-12-01

**Authors:** Myong-Song Ri, Hyon-Ju Kim, Kyong-Il Kim, Won-Il Song, Yon-Suk Jo, Kyong-Sik Ju

**Affiliations:** a High-Tech Research and Development Center, Kim Il Sung University Pyongyang Democratic People's Republic of Korea

## Abstract

Manganese dioxide (MnO_2_) nanorods were synthesized by a hydrothermal method using manganese sulfate and sodium hypochlorite as raw materials. Manganese dioxide nanorods were characterized by scanning electron microscopy (SEM), X-ray diffraction (XRD), and X-ray photoelectron spectroscopy (XPS). The as-synthesized MnO_2_ nanorods were applied to degrade rhodamine B dyes with high concentration in the presence of chlorine dioxide. The catalytic activity was the highest for manganese dioxide nanorods with rod-shaped morphology, a diameter of 80–100 nm, a length of 1.0–1.5 µm, and a crystal phase of tetragonal α-MnO_2_, prepared at 150 °C for a reaction time of 10 h. Under the reaction conditions of a chlorine dioxide concentration of 7.5 mg L^−1^, a rhodamine B concentration of 50 mg L^−1^, a reaction time of 30 min, catalyst amounts of 0.60 g L^−1^ and a pH range of 4–8, the degradation efficiency of rhodamine B approached 99.2%.

## Abstract 2

Manganese dioxide (MnO_2_) nanorods were synthesized by a hydrothermal method using manganese sulfate and sodium hypochlorite as raw materials.

## Graphical abstract

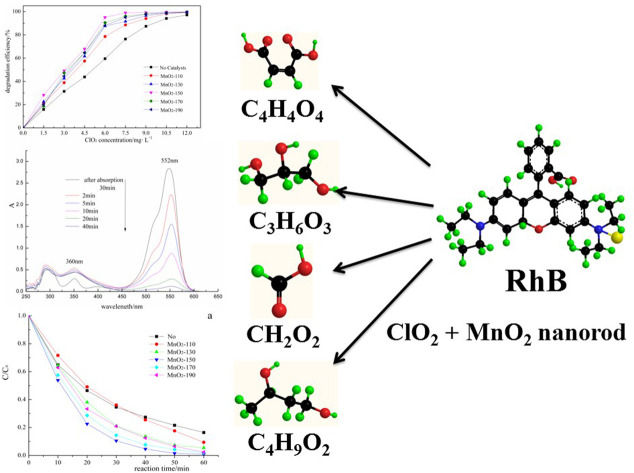

## 1 Introduction

Recently, the wastewater of organic compounds such as dyes, pigments, and phenolic compounds has become a major source of environmental pollution. These wastewater sources cause adverse effects to aquatic lives, human health and the environment.^1–4^ Various treatment techniques have been applied to remove organic compounds from wastewater, including automatic variable filtration technology (AVF), chemical oxidation, solvent extraction, membrane techniques, adsorption, advanced photo-oxidation process, coagulation, and biological treatment.^5–8^

The wet catalytic oxidation process is a mature technology used to remove toxic or non-biodegradable organic pollutants in water and is able to oxidize organic pollutants into carbon dioxide or into products that can be removed by biological treatment.^9–11^ Hydrogen peroxide,^12,34^ ozone,^13–15^ chlorine dioxide^16–18^ and peroxymonosulfate^19,20,41^ are typical oxidants that are widely used in wet catalytic oxidation processes. Here, hydrogen peroxide has the disadvantage of being an expensive and time-consuming oxidant. However, chlorine dioxide is one of the most widely used oxidants because it has strong oxidative ability, does not generate carcinogens such as trichloromethane after oxidation, and is inexpensive.^21–24^

Recently, with the rapid development of nanotechnology, the catalytic oxidation technology using nanocatalysts has been widely applied toward the treatment of organic pollutants.^25–27^ In particular, MnO_2_ nanomaterials have attracted much attention due to their low cost and high catalytic activity.^28,29^ There have been extensive reports on the removal of organic pollutants such as rhodamine B, methylene blue, sulfamethoxazole, and phenol in the presence of manganese dioxide nanomaterials as catalysts.^28–40^

MnO_2_ exists in various forms, such as α-, β-, γ-, δ- and λ-MnO_2_, according to the arrangement of octahedral units [MnO_6_] at the faces and edges of MnO_2_.^39–41^ Among them, α-MnO_2_ presented higher oxidation activity than β-MnO_2_ and γ-MnO_2_ due to its higher surface area, small pore size and strongest oxygen adsorption ability with more exposure of [MnO_6_] edges.^34–39^ In particular, it has been reported that α-MnO_2_ nanorods exhibit higher oxidative degradation properties of sulfamethoxazole than β-MnO_2_ nanorods.^40^

In the literature,^41^ it has been reported that the catalytic activity of α-MnO_2_ mainly relies on the specific surface area and crystallinity and follows the order of α-MnO_2_ nanoflowers > α-MnO_2_ nanorods > α-MnO_2_ nanoparticles > MnO_2_ microparticles. Saputra *et al.* also tested the catalytic activities of different crystallographic MnO_2_ (α, β, γ) in peroxymonosulfate solution and found that α-MnO_2_ nanowires presented the highest activity, which was attributed to the high surface area and preferable crystalline structure.^36^

Studies have been reported on the degradation of phenolic derivatives, dyes, *etc.* by ClO_2_ in the presence of catalysts, such as CuO_*x*_/Al_2_O_3_,^42^ CuO_*x*_-La_2_O_3_/Al_2_O_3_,^43,44^ NiO-CuO_*x*_-La_2_O_3_/Al_2_O_3_,^45^ Al_2_O_3_ (ref. 46) and MnO_2_-carrier.^47^ However, there have been no reports on the removal of organic pollutants by ClO_2_ in the presence of MnO_2_ nanorod catalysts.

In this paper, we synthesized MnO_2_ nanorods using manganese sulphate and sodium hypochlorite as raw materials. In addition, the catalytic performances of MnO_2_ nanorods synthesized in different temperatures were investigated in the degradation of rhodamine B by ClO_2_ as an oxidant.

## 2 Experimental

### 2.1 Catalyst synthesis

Sodium hypochlorite solution (NaClO, Aladdin, 10 wt%), manganese sulphate (MnSO_4_·H_2_O, Aladdin, 99.5%), sodium hydroxide (NaOH, Aladdin, 99.5%), and sulfuric acid (H_2_SO_4_, Aladdin, 99.5%) were used as starting materials. The ClO_2_ stock solution was stored under dark conditions at 5 °C and was standardized before using. All reagents are analytical grade and can be used without further purification. Rhodamine B (RhB) was chosen as the organic pollutant. The synthetic equation of the MnO_2_ nanorods is as follows.

1 MnSO_4_ + NaClO + H_2_O → MnO_2_ + NaCl + H_2_SO_4_

Initially, 0.02 mol of MnSO_4_ was dissolved in a 250 mL glass flask that contained 50 mL of deionized water and then 0.02 mol of NaClO solution was added with stirring. It was transferred to a 100 mL Teflon liner. The Teflon liner was loaded into a stainless steel autoclave and heated in an oven. The autoclave was heated to 150 °C and kept at this temperature for 10 h. Finally, the autoclave was cooled to room temperature naturally. The obtained precipitates were filtered, washed with distilled water and then dried in vacuum at 70 °C for 4 h.

In order to synthesize MnO_2_ nanorods with different diameters, the reaction temperature was varied from 110 °C to 190 °C, while the other reaction conditions were kept constant. The obtained catalysts were marked as MnO_2_-110, MnO_2_-130, MnO_2_-150, MnO_2_-170, and MnO_2_-190, corresponding to 110, 130, 150, 170 and 190 °C, respectively.

### 2.2 Characterization of physical properties

To study the crystallographic properties of the catalysts, XRD analysis was carried out with a D/max-RB X-ray diffractometer with the Cu Kα X-ray source at 40 kV and 100 mA. The morphology and microstructure of the catalysts were further studied by SEM (JSM-6610A, JEOL, JAPAN). The chemical bonding of the catalysts was studied by X-ray photoelectron spectroscopy (ESCALAB-250 carried out with a monochromatic Al Kα (1486.6 eV) radiation source).

### 2.3 Catalytic characterization

The degradation experiments of RhB using MnO_2_ nanorod catalysts were carried out in a 100 mL stoppered Erlenmeyer flask. Firstly, 100 mg of MnO_2_ nanorods catalyst and 10 mL of distilled water were placed in a 100 mL stoppered Erlenmeyer flask and dispersed by ultrasonic agitation for 5 min, and then 50 mL of 100 mg L^−1^ RhB solution was added and stirred for 5 min. The pH of the solution was adjusted with sodium hydroxide and sulfuric acid solution. A certain volume of 100 mg L^−1^ chlorine dioxide solution was added to the flask and the volume was calibrated with distilled water. During the reaction, the temperature of the suspension was kept at 20 ± 2 °C. At regular time intervals, 1 mL of the mixed suspension was taken by a syringe and centrifuged to separate the catalyst. At this time, 0.1 mL of 1.0 mol L^−1^ sodium thiosulfate solution was added to the sample to stop the oxidation reaction. The absorbance of the dissolved RhB solution was then measured by UV-Vis spectrophotometer at 552 nm, corresponding to the maximum absorption peak, and the degradation efficiency was calculated.

The degradation efficiency is expressed as follows:

2 *η* = (*C*_0_ − *C*) × 100/*C*_0_

where *C*_0_ is the initial concentration and *C* is the concentration measured at each time interval. In addition, RhB degradation experiments were carried out in the absence and presence of MnO_2_ nanorod catalysts.

## 3 Results and discussion

### 3.1 Physical characterization

**Fig. 1:**
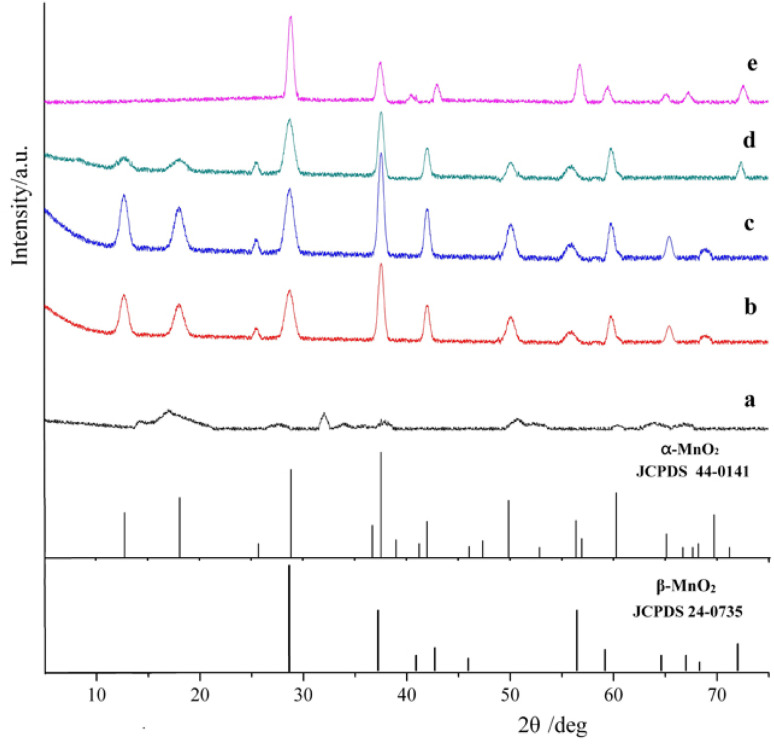
XRD patterns of the MnO_2_ nanoparticles synthesized at different temperatures. (a) 110 °C, (b) 130 °C, (c) 150 °C, (d) 170 °C, (e) 190 °C.

The phase of the as-synthesized MnO_2_ nanoparticles was characterized by XRD (Fig. 1).

The sample of manganese dioxide synthesized at 110 °C showed no significant peaks, indicating the presence of amorphous manganese dioxide (Fig. 1a). The diffraction peaks of the MnO_2_ nanorods synthesized at 130 and 150 °C appeared at 2*θ* = 12.6°, 18.0°, 25.5°,28.6°, 37.4°, 41.7°, 49.7°, 55.9°, 59.8°, 65.4°, and 68.8°, which could be indexed as the (110), (200), (220), (310), (211), (301), (411), (600), (521), (002) planes of tetragonal α-MnO_2_ (JCPDS no.44-0141), respectively (Fig. 1b and c). However, the strength increased with increasing temperature, indicating that the crystallinity increased with increasing temperature. At 170 °C, the intensity of α-MnO_2_ at 12.6°, 18.0°, 37.4° significantly decreased, indicating that α-MnO_2_ starts to transform into β-MnO_2_ (Fig. 1d). The diffraction peaks of MnO_2_ nanorods synthesized at 190 °C appeared at 2*θ* = 28.63°, 37.32°, 42.81°, 56.65°, 59.33°, and 72.4°, which could be indexed as the (110), (101), (111), (211), (220), (112) planes of tetragonal β-MnO_2_ (JCPDS no.24-735), respectively (Fig. 1e). No peaks for any impurities are observed, indicating the high purity of the synthesized product.

In general, the catalytic activity of α-MnO_2_ is higher than that of β-MnO_2_.^34–39^ Among α-MnO_2_ nanomaterials, the catalytic activity follows the order of α-MnO_2_ nanoflowers > α-MnO_2_ nanorods > α-MnO_2_ nanoparticles > MnO_2_ microparticles.^41^ Thus, the catalytic activity of manganese dioxide synthesized at 150 °C may be the highest.

**Fig. 2:**
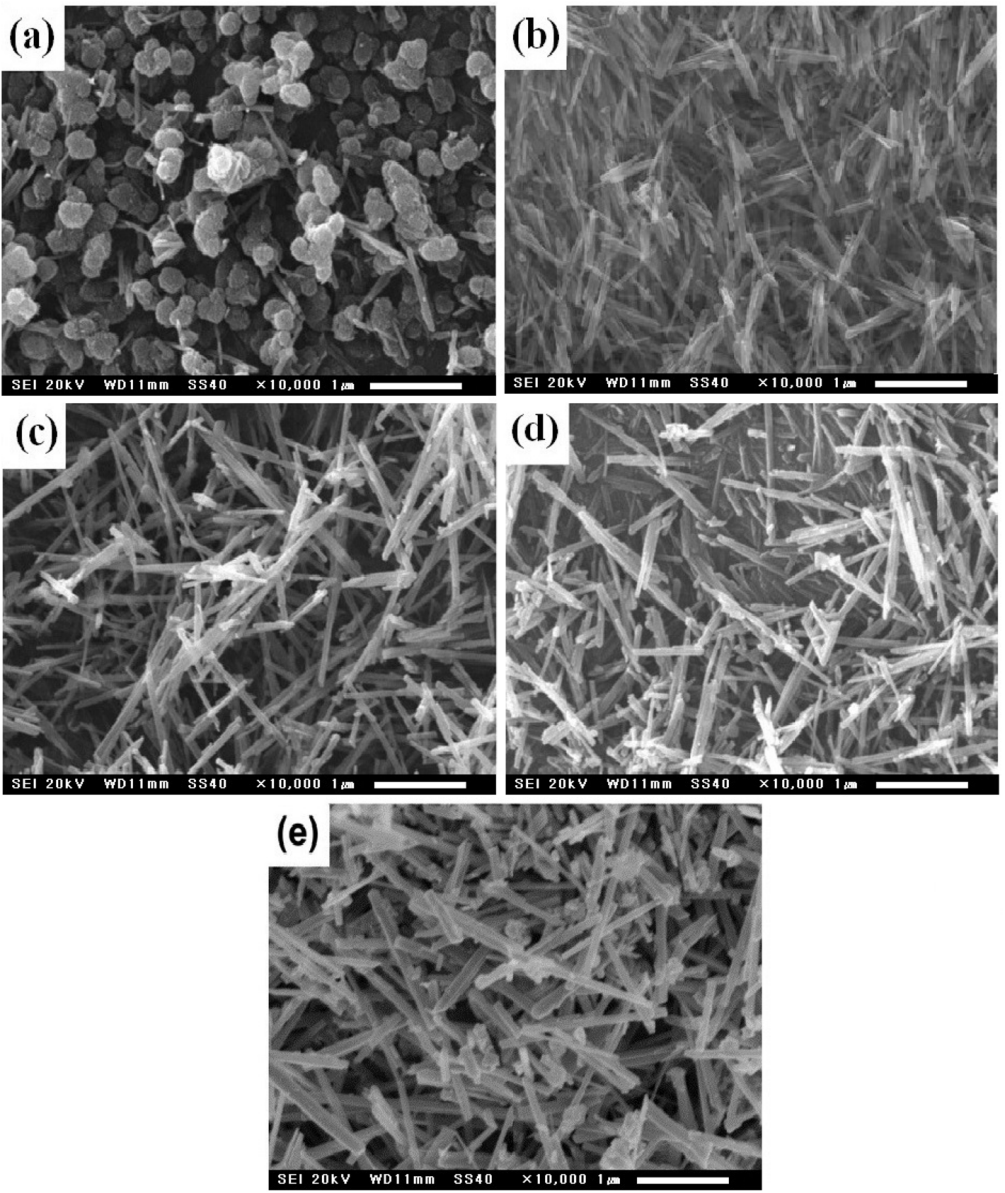
SEM images of the MnO_2_ nanoparticles synthesized at different temperatures. (a) 110 °C, (b) 130 °C, (c) 150 °C, (d) 170 °C, (e) 190 °C.

The morphologies of the as-synthesized MnO_2_ were characterized by SEM (Fig. 2).

As can be seen in Fig. 1, when the reaction temperature is lower, the obtained MnO_2_ particles are spherical. However, at higher reaction temperatures, the particles become rod-like and both their diameter and length increase. The MnO_2_ synthesized at 110 °C consists of MnO_2_ nanoparticles with a diameter of 150–200 nm and small amounts of nanorods (Fig. 2a). However, at >130 °C, MnO_2_ nanorods were synthesized. The MnO_2_ nanorods synthesized at 130–170 °C have a diameter of 70–100 nm and length of 1–1.5 µm (Fig. 2b–d). In contrast, the MnO_2_ nanorods synthesized at 190 °C have a diameter of 150–170 nm and length of 1.5–2 µm (Fig. 2e). This shows that the reaction temperature is the most important factor in MnO_2_ nanorod synthesis.

Among the manganese dioxides synthesized from 130 °C to 170 °C, the manganese dioxide synthesized at 130 °C has the largest specific surface area, while those synthesized at 150 and 170 °C have no significant differences. However, based on XRD and SEM results, it may be concluded that manganese dioxide synthesized at 130 °C has a small particle size but a low crystallinity, and the catalytic activity is lower than that of α-MnO_2_ synthesized at 150 °C.

The chemical bonding properties of the catalyst were studied by X-ray photoelectron spectroscopy. X-ray photoelectron spectroscopy is a good technology to study the relative composition of the synthesized material and the oxidation state of the manganese ion.

**Fig. 3:**
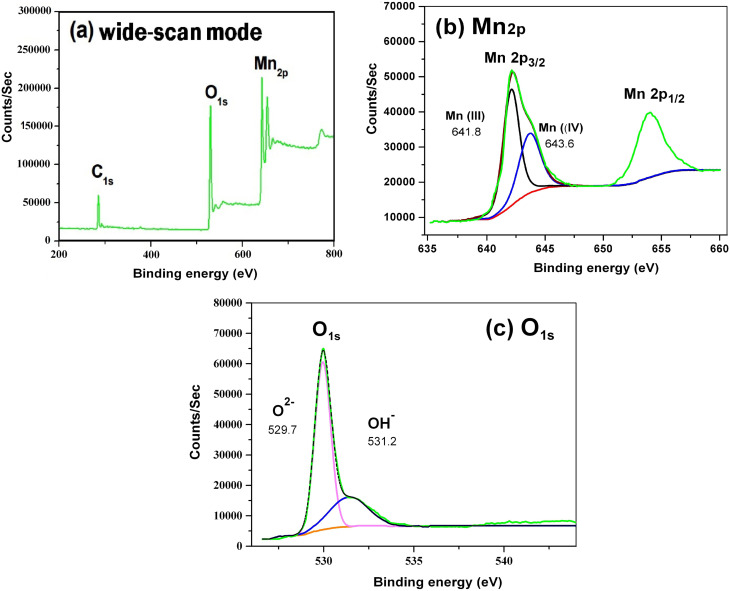
X-ray photoelectron spectra for the α-MnO_2_ nanorods (150 °C and 10 h): (a) wide-scan mode, (b) Mn 2p, and (c) O 1s.

The wide-scan X-ray photoelectron spectra, and the deconvolution of the Mn 2p and O 1s spectra of the as-prepared α-MnO_2_ are presented in Fig. 3a–c. The peaks of Mn 2p_1/2_ (654.0 eV) and Mn 2p_3/2_ (641.8 eV) have a spin energy separation of about 12.2 eV, which reveals the presence of Mn^4+^ ions in α-MnO_2_ (Fig. 3a and b).^34,49^ In the deconvolution of the Mn 2p spectrum (Fig. 3b), the Mn 2P_3/2_ peak is resolved into two components with binding energies centered at 641.8 and 643.6 eV, indicating the presence of the Mn(iii) and Mn(iv) oxidation states, respectively. The obtained binding energies match well with previous literature studies.^49,50^ The deconvolution of the O 1s spectrum resulted in two peaks at 529.7 and 531.5 eV for the α-MnO_2_ nanorod. As reported in the previous literature,^50,51^ the peak at the binding energy of 529.4–530.0 eV is assigned to lattice oxygen (in the form of O^2−^), and the peak at 531.3–531.8 eV is assigned to the surface adsorbed oxygen (such as OH). Therefore, in the case of oxygen (Fig. 3c), the two different peaks centered at 529.7 and 531.5 eV correspond to lattice oxygen (in the form of O^2−^) and surface adsorbed oxygen (such as OH), respectively.

### 3.2 Effect of several factors on the catalytic performance

In order to investigate the catalytic performance of the MnO_2_ nanorods synthesized at different temperatures, RhB was selected as the organic pollutant to be degraded in the presence of ClO_2_.

#### 3.2.1 Effect of the ClO_2_ concentration on the RhB degradation

Using the MnO_2_ nanocatalysts synthesized at different temperatures, it is very important to examine the effect on RhB degradation. The effect of the ClO_2_ concentration on the RhB degradation process using MnO_2_ nanoparticles synthesized at different temperatures was studied using the following experimental conditions: RhB concentration of 50 mg L^−1^, catalyst amounts of 0.8 g L^−1^, reaction time of 60 min, reaction temperature of 20 °C and pH 6.0.

**Fig. 4:**
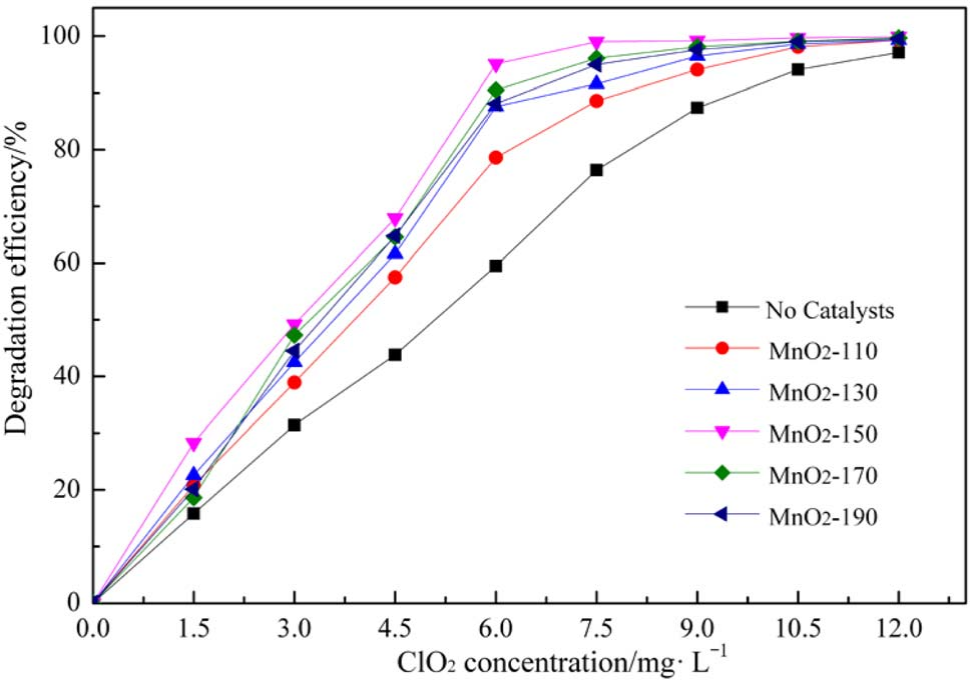
Effect of the ClO_2_ concentration in the RhB degradation process using the MnO_2_ nanoparticles synthesized at different temperatures. (reaction conditions: RhB = 50 mg L^−1^, catalysts = 0.8 g L^−1^, time = 60 min, pH = 6.0, reaction temperature 20 °C, MnO_2_-110, MnO_2_-130, MnO_2_-150, MnO_2_-170, MnO_2_-190 catalysts are MnO_2_ nanoparticles that were synthesized at 110, 130, 150, 170 and 190 °C, respectively, for 10 h).

The results clearly showed that ClO_2_ could oxidize RhB (Fig. 4). However, with the MnO_2_ nanorod catalysts, the degradation efficiency of RhB was higher than that without catalyst. In particular, in the case of the MnO_2_-150 nanorod, the degradation efficiency of RhB was highest compared to other catalysts. In the absence of the catalyst, the degradation efficiency was the lowest. This is because the MnO_2_ nanorods synthesized at 150 °C are α-MnO_2_. α-MnO_2_ can have oxidation transformation processes of Mn^4+^ → Mn^3+^ → Mn^2+^, while β-MnO_2_ only has one process of Mn^4+^ → Mn^3+^. Comparing the structures of α- and β-MnO_2_, the two-tunnel structured α-MnO_2_ will show higher activity than the single-tunnel structured β-MnO_2_ due to the greater exposure of MnO_6_ edges. This is consistent with previous literature studies^19,20^ that have reported that α-MnO_2_ exhibits higher catalytic oxidation than β-MnO_2_.

When the concentration of chlorine dioxide is 7.5 mg L^−1^ with the addition of the MnO_2_-150 catalyst, 99.0% of RhB is removed for 60 min. This is greater than the degradation efficiency (97.1%) when the chlorine dioxide concentration is 12 mg L^−1^ without the addition of a catalyst.

The effectiveness of a catalyst is that the total cost can be reduced by lowering the oxidant concentration. It also can effectively reduce the environmental pollution by residual chlorine dioxide, which can be caused by the increase of chlorine dioxide dosage. Thus, the chosen catalyst was α-MnO_2_ nanorod synthesized at 150 °C, and the optimum chlorine dioxide concentration was 7.5 mg L^−1^ with the RhB degradation efficiency above 99%.

#### 3.2.2 Effect of different amounts of catalyst

The effect of the reaction time on the RhB degradation process in the presence of different amounts of MnO_2_ nanorods (MnO_2_-150) was performed using the following experimental conditions: RhB concentration of 50 mg L^−1^, ClO_2_ concentration of 7.5 mg L^−1^, pH of 6.0, reaction temperature of 20 °C.

**Fig. 5:**
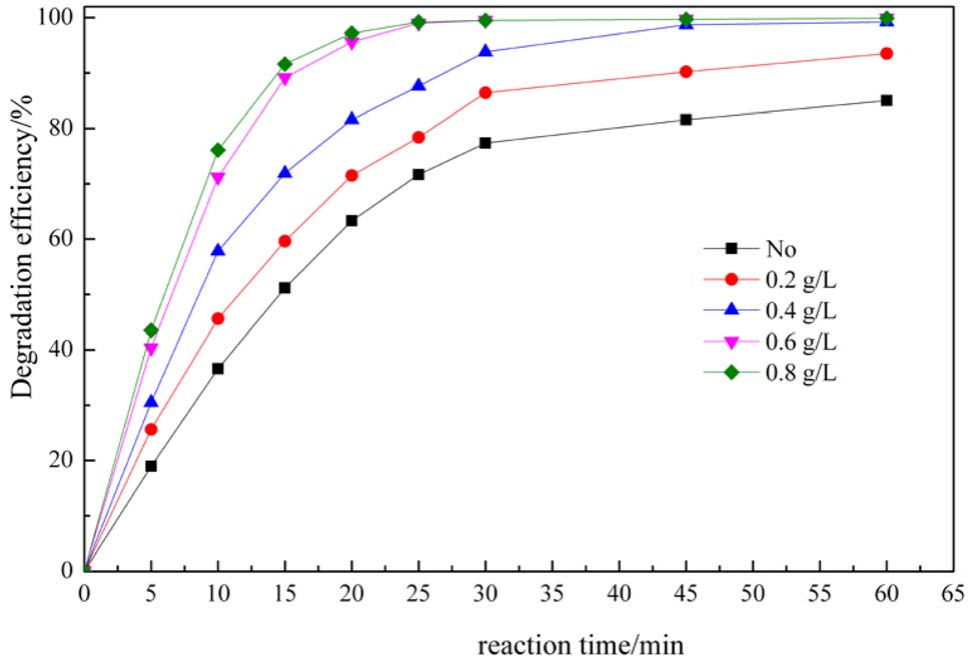
Effect of the reaction time on the degradation efficiency in the RhB degradation process with different amounts of the MnO_2_ nanorod catalyst (MnO_2_-150). (reaction conditions: RhB = 50 mg L^−1^, ClO_2_ = 7.5 mg L^−1^, pH = 6.0, and reaction temperature 20 °C).

**Fig. 6:**
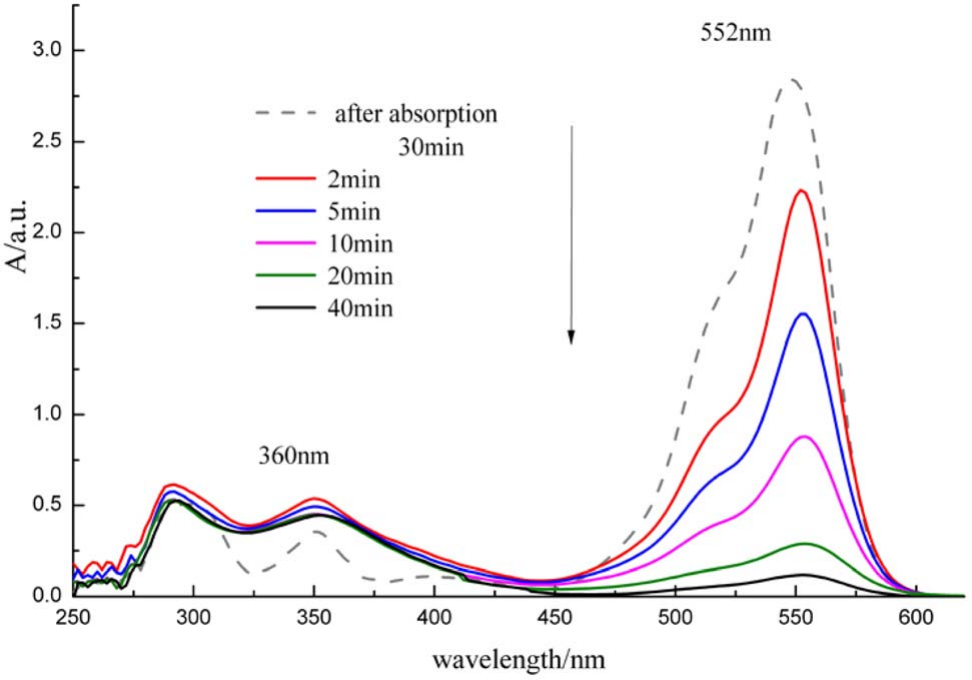
UV-Vis absorption spectra of the (RhB + ClO_2_ + MnO_2_ nanorods) solution as a function of time after ClO_2_ is added.

Fig. 5 shows the degradation efficiency of RhB as a function of the reaction time under different amounts of catalyst conditions, and Fig. 6 shows the UV-Vis absorption spectra of the (RhB + ClO_2_ + MnO_2_ nanorods) solution as a function of time after ClO_2_ is added. In the ClO_2_ catalytic oxidation process with MnO_2_ nanorods, the degradation efficiency of RhB increases rapidly with the reaction time. Also, as the catalyst amounts increased, the degradation efficiency of RhB increases. The degradation efficiency reached more than 70% after 10 min, and reached more than 99% after 30 min. Also, when the catalyst loading was higher than 0.6 g L^−1^, the degradation efficiency did not significantly increase.

As shown in Fig. 6, the maximum absorption peak of RhB at 552 nm and of ClO_2_ at 360 nm were initially observed. Upon increasing the reaction time, the maximum absorption intensity of RhB and ClO_2_ decreased. This shows that RhB was effectively removed. Therefore, the catalytic amounts and RhB degradation time were set to 0.6 g L^−1^ and 30 min, respectively. The experimental results show that the MnO_2_ nanorod catalyst not only improves the degradation rate of RhB but also shortens the reaction time, which indicates that the ClO_2_ catalytic oxidation process has more advantages in engineering applications.

#### 3.2.3 Effect of pH

The pH value is one of the most important factor in the ClO_2_ catalytic oxidation because the redox potential of chlorine dioxide and the degradation principle of MB differ with pH. The effect of pH values on the RhB degradation process was studied using the following experimental conditions: RhB concentration of 50 mg L^−1^, ClO_2_ concentration of 7.5 mg L^−1^, MnO_2_-150 catalyst amounts of 0.6 g L^−1^, reaction time of 30 min, reaction temperature of 20 °C.

**Fig. 7:**
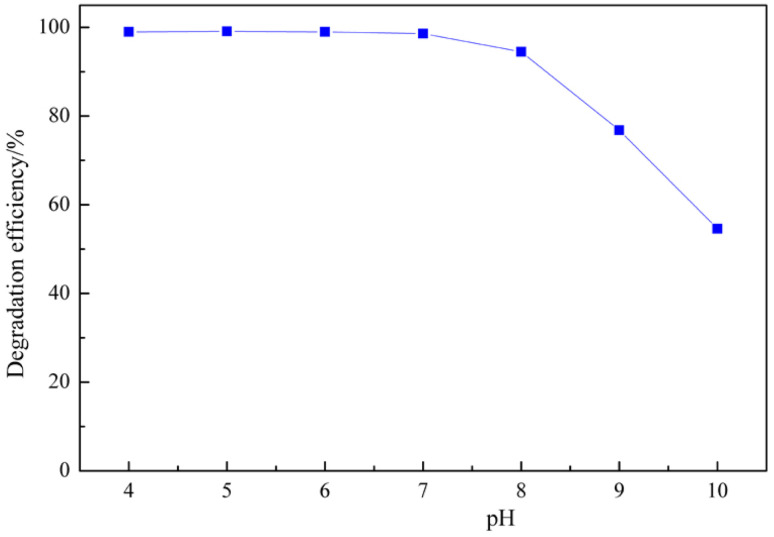
Degradation efficiency of RhB by chlorine dioxide with different pH values. (reaction conditions: RhB = 50 mg L^−1^, ClO_2_ = 7.5 mg L^−1^, MnO_2_-150 catalyst = 0.6 g L^−1^, time = 30 min, reaction temperature 20 °C).

Fig. 7 shows that the RhB degradation efficiency by ClO_2_ catalytic oxidation strongly depends on pH. When the pH value is 4–8, the degradation efficiency of RhB did not change. However, at pH > 8, the degradation efficiency is small. This is because at pH 4–8, the chemical reaction involves chlorine dioxide reacting with organic pollutants to convert chlorite ions. In contrast, at pH > 9, chlorine dioxide reacts with hydroxyl anions to form chlorite ions and chlorate ions. Chlorite and chlorate ions have a lower oxidation capacity compared to chlorine dioxide.

3 ClO_2_ + e^−^ → ClO_2_^−^ φ^θ^_ClO_2_/ClO_2__^−^ = 0.95 V

4 2ClO_2_ + 2OH^−^ → ClO_2_^−^ + ClO_3_^−^ + H_2_O (pH > 9)

However, the pH of wastewater is usually 4–9, so it is considered that it does not affect the removal of organic pollutants in real environmental conditions.

#### 3.2.4 Effect of temperature

In general, the effect of temperature on chemical reactions is one of the important factors. As the temperature increases, the rate of chemical reactions increases, which affects the overall chemical reactions. The dependence of the rate constant (*k*) on the reaction temperature can be expressed by the Arrhenius equation:

5 ln(*k*) = ln(*A*) − *E*_a_/*RT*

where *A* = frequency factor, *E*_a_ = activation energy, *R* = universal gas constant (8.315 J K^−1^ mol^−1^), and *T* = absolute temperature (K).

The effect of temperature on the RhB decomposition process was studied by varying the reaction temperature under the following conditions: RhB of 50 mg L^−1^, chlorine dioxide of 7.5 mg L^−1^, catalyst amount of 0.60 g L^−1^, and pH 6.0.

**Fig. 8:**
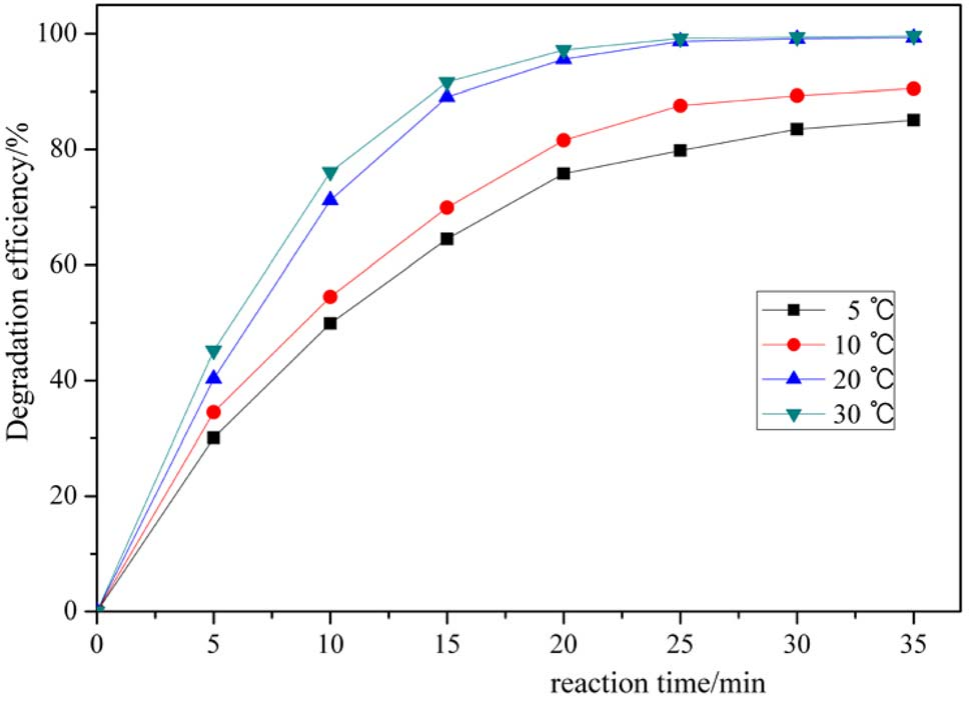
Degradation efficiency of RhB by chlorine dioxide at different reaction temperatures. (reaction conditions: RhB = 50 mg L^−1^, ClO_2_ = 7.5 mg L^−1^, MnO_2_-150 catalyst = 0.6 g L^−1^, and pH = 6.0).

As shown in Fig. 8, the degradation efficiency of RhB increases with increasing temperature. However, the degradation efficiency does not differ significantly within the reaction temperature range of 20–30 °C. At 20 °C, the degradation efficiency of RhB was 99.1% for 30 min. The reaction temperature increases, and the rate of RhB decomposition by chlorine dioxide increases. However, when the temperature is raised above 35 °C, the vapor pressure of chlorine dioxide increases, which evaporates rapidly and the decomposition rate increases. In addition, it requires a lot of energy to raise the temperature of wastewater above 30 °C. This indicates that the optimum temperature for the degradation reaction of RhB by chlorine dioxide is 20–30 °C. Therefore, the reaction temperature was chosen to be 20 °C.

### 3.3 Kinetics on the chlorine dioxide catalytic oxidation

#### 3.3.1 Degradation pathways of RhB by chlorine dioxide

**Fig. 9:**
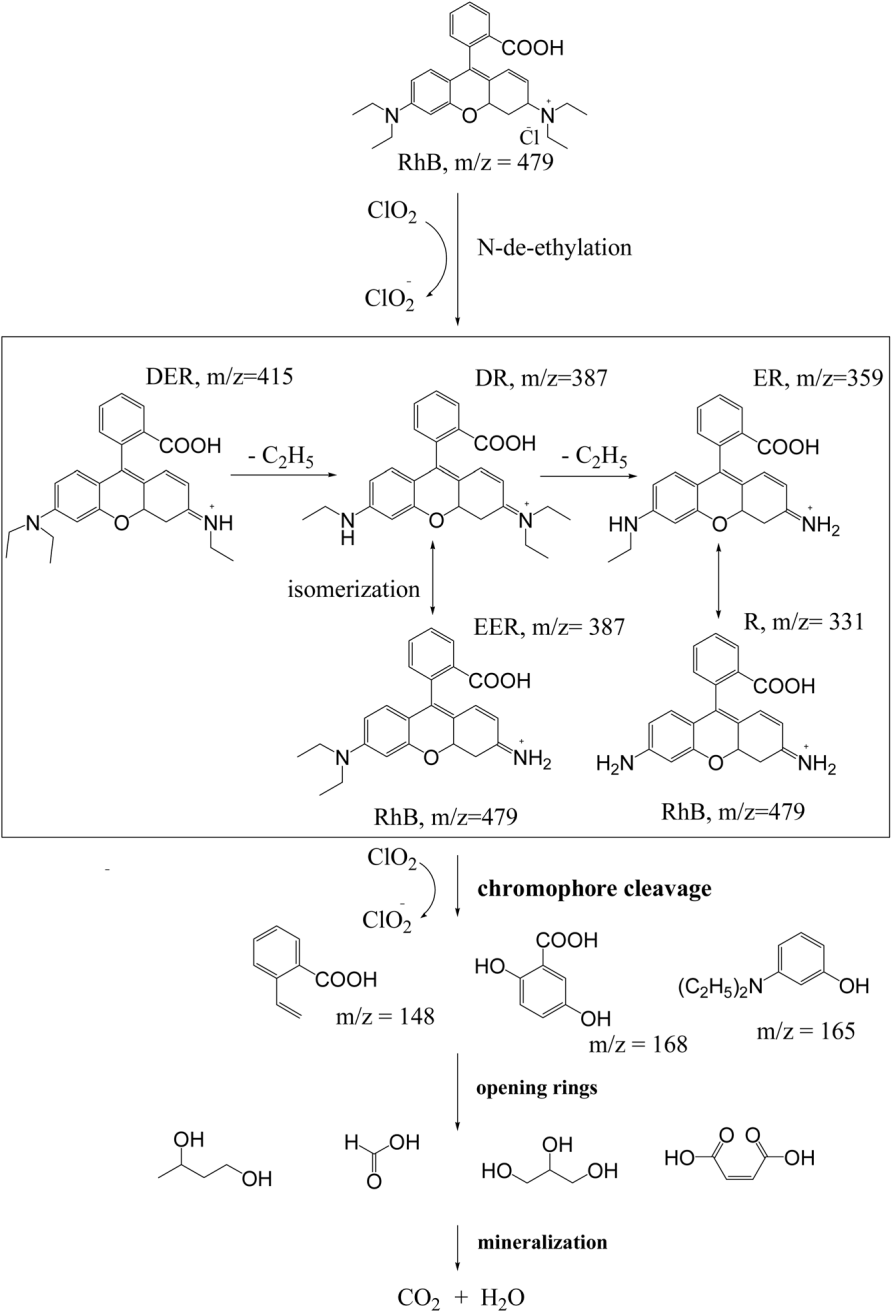
Assumed degradation pathways of RhB by chlorine dioxide.

Chlorine dioxide is mainly converted into chlorite anions and chloride anions during redox reactions.^52–54^ The assumed reaction pathways for the degradation of RhB by chlorine dioxide are shown in Fig. 9.

Chlorine dioxide is highly reactive with amine compounds, especially with tertiary amines with high electron density on nitrogen atoms.^53^ Therefore, chlorine dioxide may first attack the tertiary amine backbone on the side chain of RhB and undergoes the N-de-ethylation reaction. During the reaction, chlorine dioxide is reduced to chlorite anions.^18,54^ Then, the central carbon atom, which forms a conjugated double bond, is attacked to initiate chromophore cleavage reactions, followed by ring opening, and mineralization reactions. The oxidation products formed after the chromophore cleavage reactions may be 3-(diethylamino) phenol, 2-(2,5-dihydroxyphenyl) acetic acid and 2-vinylbenzoic acid. Then these products may be decomposed into small molecular materials such as hydroquinone, malonic acid, oxalic acid, formic acid, and gradually into mineralized products such as CO_2_ and H_2_O during the ring opening reaction.

#### 3.3.2 Kinetic study of the RhB degradation reaction

The ClO_2_ catalytic oxidation of organic compounds generally follows the kinetics of the second-order reaction.^48^ For the kinetic study of the reaction of RhB degradation by chlorine dioxide, the initial concentration of chlorine dioxide was chosen to be more than five times that of RhB. Then, the reaction rate equation is as follows.

6 d[RhB]/d*t* = −*k*_app_[ClO_2_]^*m*^[RhB]^*n*^ = −*k*′[RhB]^*n*^

Here, [ClO_2_] and [RhB] are the concentrations of chlorine dioxide and RhB, *m* and *n* are the orders with respect to the concentrations of ClO_2_ and RhB, respectively, and *k*_app_ is the apparent rate constant.

**Fig. 10:**
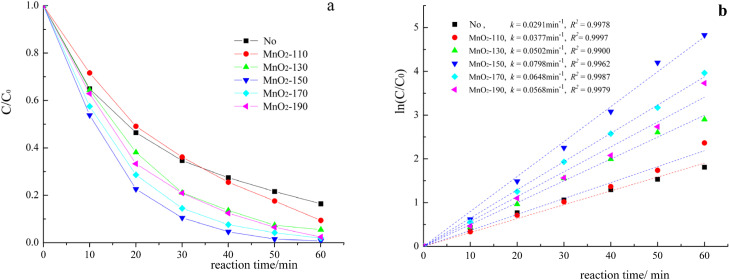
RhB degradation (a) and kinetic experiments (b) by chlorine dioxide catalytic oxidation using No (without catalyst), MnO_2_-110, MnO_2_-130, MnO_2_-150, MnO_2_-170, and MnO_2_-190 catalysts. (chlorine dioxide 7.5 mg L^−1^, RhB 50 mg L^−1^, catalyst amounts 0.60 g L^−1^, pH 6.0, and reaction temperature 20 ± 1 °C).

Kinetic studies of the RhB degradation reactions with different catalysts were carried out and the results are shown in Fig. 10.

Fig. 10a and b show the oxidative degradation of RhB using No catalyst, MnO_2_-110, MnO_2_-130, MnO_2_-150, MnO_2_-170, MnO_2_-190 catalysts in the presence of chlorine dioxide (7.5 mg L^−1^ of ClO_2_, 50 mg L^−1^ of RhB, 0.60 g L^−1^ of catalyst addition, pH 6.50). The degradation efficiency of RhB during 30 min varies according to the different catalysts: MnO_2_-150 (99.2%) > MnO_2_-170 (97.5%) > MnO_2_-190 (91.7%) > MnO_2_-130 (82.5%) > MnO_2_-110 (74.5%) (Fig. 10a). In addition, the rate constants were estimated to be 0.0291, 0.0377, 0.0502, 0.0798, 0.0648, and 0.0568 min^−1^ for the M-0(No catalyst), MnO_2_-110, MnO_2_-130, MnO_2_-150, MnO_2_-170 and MnO_2_-190 (Fig. 10b). The results indicate that the rate constants of ClO_2_ oxidation process on RhB are found to be significantly different with the catalyst, indicating that the MnO_2_ nanorods synthesized 150 °C are very effective oxidation catalysts (Table 1).

## 4 Conclusions

In this paper, the effect of MnO_2_ nanorods synthesized with manganese sulfate and sodium hypochlorite as precursors on the degradation of RhB by chlorine dioxide was studied. The characterization results showed that the morphology of MnO_2_ synthesized at 150 °C is rod shape, the diameter of MnO_2_ nanorods ranged from 80 to 100 nm and the length ranged from 1.0 to 1.5 µm, and crystal phase of tetragonal α-MnO_2_. The catalytic activity of MnO_2_ nanorods was investigated in terms of the degradation of RhB by ClO_2_ catalytic oxidation. In the degradation condition of ClO_2_ concentration 7.5 mg L^−1^, RhB concentration 50 mg L^−1^, reaction time 30 min, catalyst amounts 0.60 g L^−1^ and pH 6 ± 2, RhB degradation efficiency was up to 99.2% by the catalytic effect of the MnO_2_ nanorod. It shows that MnO_2_ nanorod is very reasonable to remove RhB in wastewater.

## Author contributions

Myong-Song Ri: investigation, writing, experiment. Hyon-Ju Kim: experiment. Kyong-Il Kim: writing – review & editing. Won-Il Song: writing – original draft. Yon-Suk Jo: formal analysis. Kyong–Sik Ju: conceptualization, methodology, investigation.

## Conflicts of interest

There are no conflicts to declare.

Appendix

**Table 1:** Characterization of catalysts for the rhodamine B degradation reaction

Catalyst	Catalyst preparation condition	Degradation condition	Degradation efficiency/%	Ref.
α-MnO_2_ nanorods	180 mg of MnCl_2_·4H_2_O, 127 mM KMnO_4_ 5 mL, 85 °C, 90 min	RhB 20 mg L^−1^ = 50 mL, 30% H_2_O_2_ = 6 mL, α-MnO_2_ = 10 mg, 10 min, pH 4–6	97.5	34
Co Fe_2_O_4_/TNTs	3.83 mmol of Co(NO_3_)_2_·6H_2_O, 7.66 mmol of Fe(NO_3_)_3_·9H_2_O, 7.66 mmol of citric acid 150 °C, 16 h	RhB = 100 mg L^−1^, oxone = 4000 mg L^−1^, catalyst = 200 mg L^−1^, pH = 10, time = 20 min	99	55
Co_3_O_4_/C	Black Co_3_O_4_-based magnetic carbonaceous nanocomposite (MCN)	RhB = 50 mg L^−1^, oxone = 250 mg L^−1^, MCN = 50 mg L^−1^, temperature = 25 °C, time = 1 h	95	56
5GO-FePO_4_ composite	A specific amount of graphite oxide, 2.29 g NH_4_H_2_PO_4_, 5.36 g Fe(NO_3_)_3_, 800 °C for 4 h	RhB = 10 mg L^−1^, H_2_O_2_ = 10 mmol L^−1^, catalyst = 1 g L^−1^, time = 120 min, pH = 2.18–10.40	96.99	57
CoFe/SBA-15	10Co9.5Fe/SBA-15-700	RhB = 5.0 mg L^−1^, PMS/RhB molar ratio = 20 : 1, catalyst = 0.10 g L^−1^, temperature = 25 °C, time = 2 h	95	58
Cu/Al_2_O_3_/g-C_3_N_4_ composite	Cu_12_/Al_2_O_3_/C_3_N_4_	RhB = 20 mg L^−1^, H_2_O_2_ = 10 mmol L^−1^, catalyst = 1.0 g L^−1^, temperature = 25 °C, pH = 4.9, time = 2 h	96.4	59
Fe_2_O_3_-Kaolin	Kaolin 2 g, 0.2 mol L^−1^ Na_2_CO_3_, 0.4 mol L^−1^ Fe(NO_3_)_3_, 400 °C, 3 h	RhB = 15.0 mg L^−1^, H_2_O_2_ = 0.05 mol L^−1^, catalyst = 1.0 g L^−1^, pH = 2.21, time = 120 min	98	60
Cu-dopedLaTiO_3_	LaTi_0.4_Cu_0.6_O_3_	RhB = 8 mg L^−1^, H_2_O_2_ = 0.02 mol L^−1^, catalyst = 1.4 g L^−1^, time = 120 min	94	61
CNTs	Lengths 0.5–2 µm diameters 30–50 nm	RhB = 20 mg L^−1^, PS = 119.0 mg L^−1^, CNT = 0.2 g L^−1^, pH = 3–9 time = 150 min	100	62
Ag@CuO nanocomposite	1.0 g Cu_2_(OH)_2_CO_3_, 0.5 g silver nitrate, 20 mL water, 300 °C for 24 h	RhB = 10 mg L^−1^, PS = 200 mg L^−1^, Ag@CuO = 0.5 g L^−1^, DMPO = 100 mmol L^−1^, time = 30 min	98	63
SDS@Fe_3_O_4_	FeCl_3_·6H_2_O (0.40 M), FeCl_2_·4H_2_O (0.20 M), SDS (0.10 M),300 mL of DI water, 200 mL of 25% NH_4_OH	RhB = 10 mg L^−1^, H_2_O_2_ = 2.0 × 10^−1^ mol L^−1^, SDS@Fe_3_O_4_ = 0.1% w/v, pH = 3, temperature = 25 ± 2 °C		64
α-MnO_2_ nanorods	Diameter of nanorods: 80–100 nm, length of nanorods: 1.0 to 1.5 µm	RhB = 50 mg L^−1^, ClO_2_ = 7.5 mg L^−1^, α-MnO_2_ = 0.6 g L^−1^, time = 40 min, pH = 4–9	99.7	This study

## Data Availability

The data that support the findings of this study are available from the corresponding authors upon reasonable request. The results presented in the study have been obtained by using the chlorine dioxide catalytic oxidation experiments.
